# Self-Reported Occupational Injuries and Perceived Occupational Health Problems among Latino Immigrant Swine Confinement Workers in Missouri

**DOI:** 10.1155/2018/8710901

**Published:** 2018-06-19

**Authors:** Athena K. Ramos, Axel Fuentes, Marcela Carvajal-Suarez

**Affiliations:** ^1^Center for Reducing Health Disparities, College of Public Health, University of Nebraska Medical Center, 984340 Nebraska Medical Center, Omaha, NE 68198-4340, USA; ^2^Rural Community Workers Alliance, 110 E. 3rd St., Milan, MO 63556, USA

## Abstract

Swine production has changed dramatically, and in the United States production often takes place in concentrated animal feeding operations (CAFOs). Because of the size and density of these types of facilities, workers may be exposed to serious occupational health risks such as noxious gases, agricultural dusts, elevated noise levels, and zoonotic diseases. This descriptive study examines self-reported occupational injuries and perceived occupational health problems among a convenience sample of 40 Latino immigrant swine confinement workers (92.5% male;* M* age = 36.1 years;* SD* = 10.0) in Missouri. Results indicated that seventeen workers (42.5%) rated their health as fair or poor, thirteen (32.5%) had experienced an occupational injury, and eleven (28.2%) reported occupational health problems such as burning eyes, muscular pain, headaches, coughing, nausea, nasal congestion, and sneezing. The majority of workers did not perceive their job to be dangerous. Clearly, more must be done to protect workers, especially immigrant workers, who may not have the same access to information, training, or other protections. Health and safety should be a priority for both farmworkers and farm employers. Practical and policy-based implications and recommendations are discussed.

## 1. Introduction

The United States is the second largest producer of pork tonnage in the world [1]. To meet the growing global demand for meat, significant structural changes in the swine production industry have taken place over the last few decades in USA [2, 3]. Many farms now are specialized in specific phases of production, are vertically integrated, and are corporately owned [4, 5]. Because of these changes, there has been an increase in concentrated animal feeding operations (CAFOs) throughout USA defined as “where animals are kept and raised in confined situations ([6], n.p.).” This type of animal production was first developed in USA in the 1950s and spread globally in the 1990s [7]. Currently, most of the swine CAFOs are located in the upper Midwest and Southeastern United States [1, 7]. These changes in production facilities and processes have created a new occupational environment, which has resulted in a need for more hired labor, often filled by immigrant workers [8].

Immigrants may be vulnerable workers due to their immigration legal status, the contingent nature of their employment, and their mobility [9, 10]. Immigrant workers may also have lower levels of formal education and have limited English proficiency (LEP), which may restrict their access and knowledge of occupational health and safety information, labor rights, and training opportunities [11]. A previous study of immigrant farmworkers highlighted that workers who were proficient in English were significantly more likely to have received job-related training than those who had LEP [12]. As more and more immigrants fill agricultural jobs, it will become increasingly important to ensure that these workers have access to culturally and linguistically appropriate job-related health and safety information, proper personal protective equipment, adequate training opportunities, and a regular source for healthcare services.

Years of research have shown that working in agriculture can be dangerous. The agriculture, forestry, and fishing sector consistently has a high rate of both nonfatal and fatal occupational injuries [13]. Farmworkers are exposed to chemicals, machinery, animals, repetitive motions, extreme weather, and long work hours, which may increase their risk for occupational injury and both acute and long-term health problems [14, 15]. They may have high levels of stress and lack adequate healthcare services [16, 17]. Due to the size and density of CAFOs, workers may be exposed to additional health and safety risks such as high levels of noxious gases (e.g., ammonia, methane, carbon dioxide, and hydrogen sulfide), agricultural dusts, and elevated noise levels [5, 14, 18–22]. A specific concern with CAFO facilities is the potential capacity for the evolution and spread of novel diseases due to limited air spaces and waste removal practices [7, 23]. Among swine production workers, these exposures may result in chronic and acute respiratory conditions such as asthma, bronchitis, chronic obstructive pulmonary disease (COPD), coughing, hypersensitivity pneumonitis, throat irritation, sinus problems such as sneezing, noise-induced hearing loss (NIHL), and increased missed work days [5, 18, 19, 24].

Few studies have explored swine confinement worker health as reported by the workers themselves [5, 20, 25]. Although the swine production industry is dependent on an immigrant workforce [26], little is known about the health and well-being of this important, yet vulnerable, workforce. In order to better protect immigrant animal confinement workers from occupational injuries or related health problems, we must first understand their experiences and perceptions. Therefore, the purpose of this study is to describe self-reported occupational injuries and perceived occupational health problems among Latino immigrant swine confinement workers.

## 2. Methods

Although the data presented are cross-sectional, they are part of prospective cohort pilot study that addressed health and well-being among Latino immigrant swine confinement workers and another adult who lived in the same household.

### 2.1. Participants

According to the US Bureau of Labor Statistics, a total of 1,939 people were employed in hog and pig farming in Missouri in 2015 (North American Industry Classification System [NAICS] code 1122) [27]. Although the total number of Latino immigrant workers in swine confinement in Missouri is not available, a small cohort of 40 Latino immigrant swine confinement workers was recruited to participate in this study. To be eligible to participate in this study, workers had to be at least 18 years old (the age of majority in the state of Missouri), be an immigrant of Hispanic/Latino descent, and currently work in a swine CAFO in Missouri. Workers were employed at various confinement facilities throughout the state and were recruited through convenience sampling methods in Audrain, Linn, and Sullivan counties.

### 2.2. Procedures

This study was developed through a community-engaged research partnership between the University of Nebraska Medical Center (UNMC), Center for Reducing Health Disparities, and the Rural Community Workers Alliance (RCWA), a worker advocacy nonprofit organization in Missouri. First, study procedures and the rights of research participants were explained to each participant and informed consent was obtained. Verbal face-to-face interviews were conducted with workers. Interviews were based on a standardized questionnaire consisting of valid and reliable measures to the extent possible. Questions addressed occupational context, physical health, emotional health, stress, and demographics. All study materials were available in English and Spanish. Interviewers were bilingual and bicultural; therefore, participants had the option to participate in either language. All interviews were conducted in Spanish except for one participant who chose to respond in English. Interviews lasted approximately 45 minutes and were conducted at participants' homes or in the RCWA office. Participants were given a $10 gift card for their participation. All data was collected between June and August 2015. The study was approved by University of Nebraska Medical Center's Institutional Review Board.

### 2.3. Measures

#### 2.3.1. Self-Rated Health

General health status was measured using a single question related to self-rated health, “Would you say that in general your health is…excellent, very good, good, fair, or poor?” Response options were coded 4, 3, 2, 1, and 0, respectively. This question has been consistently used on various population health surveys and was found to be useful in predicting objective measures of various health outcomes including morbidity and mortality [28–30]. Self-rated health was dichotomized for part of the analysis based on standard conventions, fair and poor (0) and good, very good, and excellent (1).

#### 2.3.2. Occupational Injury

Occupational injury was assessed with a single question referring to the worker's current employment, “Have you ever been injured on the job?” Response options were either yes (1) or no (0). If a participant responded that they had been injured on the job, then a series of questions about the injury followed, such as type of injury, part of the body injured, source of the injury, and amount of lost time due to the injury. Finally, participants were asked if they knew any other workers who had been injured on the job, yes (1) or no (0).

#### 2.3.3. Occupational Health Problems Perceived to Be due to Working with Swine

Participants were asked if they believed that they had health problems as a result of working with swine. Response options were either yes (1) or no (0). Participants who responded “yes” were then asked what types of occupational health problems they experienced such as allergies, respiratory problems, burning eyes, headaches, hearing problems, sinus problems, infections, muscular pain, nausea, skin disorders, throat irritation, or others. Participants were also asked to disclose their smoking status, either not a smoker (0) or current smoker, which included both daily and nondaily smokers (1).

#### 2.3.4. Job Context

Job context was assessed using a series of continuous variables including number of hours worked per week, number of animals in the CAFO, and number of animals a worker personally cared for in the CAFO. Additionally, participants were asked “how dangerous do you feel your job is?” to assess perceived occupational risk. Response options included not at all dangerous (0), a little dangerous (1), dangerous (2), or very dangerous (3).

#### 2.3.5. Demographic Covariates

Age, length of employment, education, English-language proficiency, and relationship status were used as covariates. Age was recorded as a continuous variable; however, age categories were also created, 18-24, 25-40, or over 41 years of age. Length of employment was categorized into less than 1 year, 1-3 years, or more than 3 years tenure at the current employer. Education level was assessed by a single question, “What is the highest grade or year of school you completed?” There were six categorical response options: (1) never attended school, (2) elementary (grades 1-8), (3) some high school (grades 9-11), (4) high school graduate/GED, (5) some college or technical training, and (6) college graduate or higher. In part of this analysis, education was dichotomized into completed less than a high school education (0) or completed a high school education or more (1). English-language proficiency was assessed using a single question, “How well do you speak English?” Response options included not at all (0), not very well (1), well (2), and very well (3). Responses were dichotomized according to standard procedures into LEP for those who responded that they do not speak English at all or not very well and proficient in English for those who responded that they speak English well or very well [31]. Relationship status was categorical and options included married, member of unmarried couple, and never married.

### 2.4. Data Analysis

SPSS version 23.0 was used to analyze the data. Descriptive statistics including frequencies, means, and standard deviations were calculated. Pearson's correlations were used to assess the strength of the relationships between study variables and covariates that were hypothesized to be related to occupational injury and job-related health problems, and a standard* p* value of .05 was used to determine significance.

## 3. Results

Of the 40 workers who participated in the study, the sample was mainly male (92.5%) and was 36 years old on average. Twenty-eight participants were from Mexico and 12 were from Central America. As can be seen from Table 1, most had low levels of formal education completion. Most workers were relatively new to the swine production industry, and 34 workers (85%) had been employed at their current employer for less than three years.

Participants worked an average of 53 hours per week, but working hours ranged between 40 and 90 hours per week. Anecdotally, some participants mentioned during the interview that their regular schedule was to work 13 days straight and then have one day off. Facilities ranged in size from housing 2,800 swine to 80,000 swine. On average, workers were responsible for caring for 2,890 animals each.

Seventeen workers (42.5%) rated their health as fair or poor, and only half of the sample had health insurance. Twenty-six workers (66.7%) did not have a regular healthcare provider and twelve could not afford to see a doctor within the past 12 months, thereby forgoing needed treatment (Table 2).

Thirteen workers had experienced an occupational injury, and, of these, the majority said that an animal was the source of their injury. Five of the 13 workers who had been injured lost productive time from work due to their injuries.  Table 3 provides details about the occupational injuries and health problems experienced by participants.

Only thirteen participants (32.5%) responded that their job was dangerous or very dangerous; however, more than half of the sample responded that they knew another worker who had been injured on the job. As highlighted in Table 4, reporting an occupational injury was significantly positively associated with knowing another worker who had been injured (*r* = .45 and* p* < .01). Age was significantly positively associated with knowing another other worker who had been injured (*r* = .49 and* p* < .01) and significantly negatively associated with English-language proficiency (*r* = -.47 and* p* < .01).

Finally, being a current smoker was significantly positively associated with reporting allergies (*r* = .57 and* p* < .05). As length of employment increased, reports of coughing also increased (*r* = .80 and* p* < .01).

## 4. Discussion

Few studies have explored immigrant swine confinement worker health and safety in the Midwest. This study sought to describe occupational injuries and health problems among a small cohort of Latino immigrant swine CAFO workers. We found that participants worked long hours and were responsible for a large number of animals. Nearly, one-third of workers had experienced an occupational injury, mainly due to animal handling. Many workers reported occupational health problems associated with working in a CAFO including burning eyes, muscular pain, headaches, coughing, nausea, nasal congestion, and sneezing. Consistent with previous studies, being a current smoker increased the risk of reporting occupational allergies [20], and the longer a person worked at the CAFO, the more likely they were to report coughing [5, 18, 19, 24, 32]. Clearly, there is a need to improve the work environment within swine CAFOs to improve worker perceived health and reduce the potential for occupational injuries.

The first step in preventing occupational injuries and health problems is to understand the current context of the work environment. Then, workers, employers, and government entities can work together to implement strategies to reduce such injuries and health problems. Missouri and other major swine producing states could implement health and safety strategies across the hierarchy of controls, a framework to reduce and eliminate work-related hazards [33, 34]. Based on the framework, the most effective strategy is to physically remove the hazard followed by substituting out the hazard. Oftentimes, such changes are not feasible. Therefore, following lower levels of the hierarchy of controls including engineering controls that are built into the confinement facility, providing employee training, changing work practices and behaviors, and ensuring the provision and use of personal protective equipment may be the most practical strategies for reducing occupational injuries. The following discussion will focus primarily on practical work-related implications from this study such as providing training, changing work practices and behaviors, and ensuring the provision and use of personal protective equipment as well as modifying the healthcare system and assisting providers to better identify occupational health concerns.

Workers have a right to know about the hazards in their workplace per the OSHA Hazard Communication Standard, 29 CFR 1910.1200 [35], but most participants in this study did not recognize the risks. By having job-related safety and health information available, a worker or potential worker can make informed decisions about their livelihood and whether or not to accept the level of risk associated with particular employment. Workers have a right to safety and health training and instruction. According to the International Labour Organization's C-184 Safety and Health in Agriculture Convention, employers shall “ensure that adequate and appropriate training and comprehensible instructions on safety and health and any necessary guidance or supervision are provided to workers…including information on the hazards and risks associated with their work and the action to be taken for their protection, taking into account their level of education and differences in language” [36, 37]. Therefore, all agricultural employers should provide health and safety training to employees at the time of hire and at regular intervals throughout their employment in a language that is understandable to the workers. Training is particularly important for new employees, many of which do not have prior experience working with swine. Such training should address specific animal handling strategies, since animals were the primary source of injury but may also include first aid, confined space entry, and equipment handling techniques. Scheduling regular safety discussions may also highlight farm management's commitment to safety and worker health.

Occupational injuries are often underreported, particularly those experienced by immigrant workers due to fear of reporting and working without legal status, lack of knowledge of how to report injuries, and the financial impact of being unable to work. All workers should know how to report an injury and should be encouraged to report without having to fear retribution. “Near miss” events, those that did not result in injury or illness but had the potential to, should also be recorded [38]. Understanding near miss risks may help both workers and management to develop stronger safety systems, improve hazard controls, and reduce job-related risks over time.

Because respiratory health issues are a primary health concern in swine production facilities and among workers in this study, farms should be encouraged to have a respiratory protection program in place. This may help improve the health of workers by reducing exposure to dusts, gases, and zoonotic diseases but may also have secondary effects such as improving animal growth [22]. Employers may also consider preemployment screenings to assess respiratory health so that workers can be assigned tasks that will not exacerbate any preexisting health conditions. Ongoing health screening may also help protect workers by monitoring any changes in their health. All workers who smoke should be encouraged to quit due to the potential synergistic respiratory and allergic responses that may be triggered by exposures within the CAFO environment.

Personal protective equipment is the first line of defense against hazards for most workers. Workers need to understand how to use and have access to appropriate personal protective equipment such as respirators, hearing protection, and goggles and be encouraged to use it consistently when exposed to hazards [12, 21].

For workers who can access healthcare services, better systems are needed to be able to identify job-related hazards and health concerns [39]. Occupation should be incorporated into electronic medical record systems and part of a routine in-take procedures. Healthcare providers, especially those in rural areas, need to be educated on occupational exposures associated with agriculture, and specific agricultural medicine courses are available throughout USA. Without such training, workers may be misdiagnosed or not receive appropriate treatment for occupation-related health concerns.

Swine production is an important economic driver for the state of Missouri and in 2015 resulted in $939 million in cash receipts [40]. To maintain the economic vitality of the industry, improving health and safety is necessary. This may help to reduce insurance claims and lost productivity. One policy implication that follows from this study is that the state of Missouri may consider mandating that farm employers provide workers' compensation to ensure that all workers have some sort of medical and/or disability coverage if an occupational injury or illness occurs. Currently, farm employers have the option to provide this coverage but are not required to do so [41]. As highlighted in this study, many of the injuries that occurred are serious and required extended periods of lost work time, which may have affected these workers' quality of life. Undoubtedly, more can be done to protect immigrant swine confinement workers.

### 4.1. Limitations

This study had a number of limitations to note. First, this study had a small sample size and used a cross-sectional design; therefore, it is not possible to determine causality. Since this study focused specifically on Latino immigrant swine CAFO workers, it is not possible to draw conclusions about swine CAFO workers of different racial or ethnic backgrounds or agricultural workers in other production sectors. Although trained interviewers conducted the interviews with workers, participant responses were subject to social desirability bias and caution should be used when interpreting these results. Cultural connotations of strength and masculinity (e.g., machismo) may have played a role in potential underreporting of injuries and health problems. Next, unhealthy individuals may have chosen not to work in swine confinement because it may exacerbate their health issues; therefore, there could be a selection bias based on the healthy worker effect [18, 19]. Finally, data were not corroborated with employer reports of occupational injury or illness.

## 5. Conclusion

Farmworker health is a social justice and public health concern. This study found high rates of self-reported occupational injuries and perceived job-related health problems among Latino immigrant swine confinement workers; however, there was low awareness among these workers of the risks associated with working in a swine confinement facility. Because this was not an intervention study, our results only provide a limited characterization of the situation. However, by understanding the workers' perspective, agricultural health and safety specialists and industrial hygiene professionals may be able to develop relevant and effective control strategies and training initiatives. Future research should explore objective measurements of various occupationally related health conditions among immigrant farmworkers, incorporate employer reports of injury and associated workers' compensation costs, and use longitudinal designs that incorporate both subjective and objective measures to assess the health impact of working in a swine CAFO over time.

## Figures and Tables

**Table 1:** Demographic characteristics of participants (*N* = 40).

Variables	*N* (%)	M (SD)
Age		36.1 (10.0)
18-24 years	5 (12.8)	
25-40 years	22 (56.4)	
Over 41 years	12 (30.8)	
Length of employment at current production facility		
Employed for less than 1 year	17 (42.5)	
Employed for 1-3 years	17 (42.5)	
Employed for more than 3 years	6 (15.0)	
Number of hours worked per week		52.7 (10.4)
40-45 hours/week	13 (33.3)	
46-60 hours/week	20 (51.3)	
More than 61 hours/week	6 (15.4)	
Number of animals personally cared for		2890.2 (3476.7)
1-1000 swine	12 (41.4)	
1001-3000 swine	9 (31.0)	
More than 3001 swine	8 (27.6)	
Education		
Completed less than high school	31 (77.5)	
Completed high school or higher	9 (22.5)	
English language proficiency		
Limited english proficiency	32 (80.0)	
Proficient in English	8 (20.0)	
Relationship status		
Married	22 (56.4)	
Member of unmarried couple	12 (30.8)	
Never married	5 (12.8)	

Note: total number in each category may not equal total number of participants due to missing data.

**Table 2:** Health and healthcare access characteristics of participants (*N* = 40).

Variables	*N* (%)
Health status	
Good health (excellent, very good, or good self-rated health)	23 (57.5)
Poor health (fair or poor self-rated health)	17 (42.5)
Has health insurance coverage	20 (51.3)
Has a regular healthcare provider	13 (33.3)
Unable to seek care in last 12 months due to cost	12 (31.6)
Current smoker	7 (17.5)

**Table 3:** Occupational injuries and health problems reported by participants (*N* = 40).

Variables	*N* (%)
Experienced an occupational injury	13 (32.5)
Knows other workers who have been injured	21 (52.5)
Major types of injury (*N* = 13)	
Broken bone	3 (23.1)
Inhalation	3 (23.1)
Accidental injection/needle stab	3 (23.1)
Muscle sprain or strain	3 (23.1)
Part of body injured	
Leg, knee, or hip	4 (30.8)
Hand or wrist	3 (23.1)
Eyes	2 (15.4)
Head or neck	1 (7.7)
Back	1 (7.7)
Arms or shoulders	1 (7.7)
Fingers	1 (7.7)
Cause of injury	
Animal	10 (76.9)
Machine	2 (15.4)
Other	1 (7.7)
Lost time due to injury	
No time lost	8 (61.5)
2-6 days lost	1 (7.7)
More than 30 days lost	4 (30.8)
Have health problems due to working with hogs	11 (28.2)
Type of occupational health problems	
Allergies	3 (7.5)
Asthma	2 (5.0)
Bronchitis	2 (5.0)
Coughing	7 (17.5)
Eyes burning	10 (25.0)
Headaches	7 (17.5)
Hearing problems	2 (5.0)
Infections	1 (2.5)
Muscular pain	9 (22.5)
Nausea	4 (10.0)
Nasal congestion	4 (10.0)
Sneezing	6 (15.0)
Skin disorders	1 (2.5)
Pain in throat	3 (7.5)

Note: total number in each category may not equal total number of participants who reported an injury or health problem. Multiple health problems may have been reported by a worker and each health concern is represented in the table.

**Table 4:** Correlations between study variables.

	Occupational injury	Number of hours worked per week	Number of animals personally cared for	Know other workers who have been injured	Age	English language proficiency	Education
Occupational injury	-						
Number of hours worked per week	.11	-					
Number of animals personally cared for	-.14	.23	-				
Know other workers who have been injured	.45^*∗∗*^	.16	.12	-			
Age	.31	.05	-.08	.49^*∗∗*^	-		
English language proficiency	-.30	.03	.46^*∗*^	-.03	-.47^*∗∗*^	-	
Education	.01	.21	.29	-.09	-.21	.30	-

*∗* indicates *p* < .05; *∗∗* indicates *p* < .01.
